# In vitro and in vivo activities of the carbonic anhydrase inhibitor, dorzolamide, against vancomycin-resistant enterococci

**DOI:** 10.7717/peerj.11059

**Published:** 2021-03-30

**Authors:** Nader S. Abutaleb, Ahmed E.M. Elhassanny, Daniel P. Flaherty, Mohamed N. Seleem

**Affiliations:** 1 Department of Biomedical Sciences and Pathobiology, Virginia-Maryland College of Veterinary Medicine, Virginia Polytechnic Institute and State University (Virginia Tech), Blacksburg, VA, United States of America; 2 Department of Comparative Pathobiology, College of Veterinary Medicine, Purdue University, West Lafayette, IN, United States of America; 3 Department of Medicinal Chemistry and Molecular Pharmacology, College of Pharmacy, Purdue University, West Lafayette, IN, United States of America; 4 Purdue Institute for Drug Discovery, Purdue University, West Lafayette, IN, United States of America

**Keywords:** Carbonic anhydrase inhibitors, Vancomycin-resistant enterococci (VRE), Antibiotics resistance, VRE decolonizing agents

## Abstract

Vancomycin-resistant enterococci (VRE) are a serious public health threat and a leading cause of healthcare-associated infections. Bacterial resistance to antibiotics recommended for the treatment of enterococcal infections complicates the management of these infections. Hence, there is a critical need for the discovery of new anti-VRE agents. We previously reported carbonic anhydrase inhibitors (CAIs) as new potent VRE inhibitors. In the present study, the activity of the CAI, dorzolamide was evaluated against VRE both in vitro and in vivo. Dorzolamide exhibited potent activity against a panel of clinical VRE isolates, with minimum inhibitory concentration (MIC) values ranging from 1 µg/mL to 8 µg/mL. A killing kinetics experiment determined that dorzolamide exhibited a bacteriostatic effect against VRE, which was similar to the drug of choice (linezolid). Dorzolamide interacted synergistically with gentamicin against four strains of VRE, and exhibited an additive interaction with gentamicin against six VRE strains, reducing gentamicin’s MIC by several folds. Moreover, dorzolamide outperformed linezolid in an in vivo VRE colonization reduction mouse model. Dorzolamide significantly reduced the VRE burden in fecal samples of mice by 2.9-log_10_ (99.9%) and 3.86-log_10_ (99.99%) after 3 and 5 days of treatment, respectively. Furthermore, dorzolamide reduced the VRE count in the cecal (1.74-log_10_ (98.2%) reduction) and ileal contents (1.5-log_10_ (96.3%)) of mice, which was superior to linezolid. Collectively, these results indicate that dorzolamide represents a promising treatment option that warrants consideration as a supplement to current therapeutics used for VRE infections.

## Introduction

Vancomycin-resistant enterococci (VRE) infections are a major challenge globally and require the development of new therapeutics. Prolonged hospitalizations can lead to colonization of the gastrointestinal tract (GIT) by VRE, which in turn can result in life-threating infections, such as endocarditis, systemic infections, and urinary tract infections (UTI) (Cetinkaya, Falk & Mayhall, 2000). In addition, VRE infections are associated with increased rates of mortality as well as high economic burden due to extended periods of hospitalizations (Stagliano et al., 2017). The U.S. Centers for Disease Control and Prevention (CDC) recently reported that VRE infections contributed to nearly 55,000 hospitalizations in the United States in 2017, which resulted in a 10% mortality rate and cost nearly $540 million in healthcare costs (Abutaleb & Seleem, 2020c).

The lack of effective treatment options for VRE infections has created a serious need for the development of new, effective anti-VRE therapeutics. Currently, linezolid is the only drug approved by the U.S. Food and Drug Administration (FDA) for the treatment of VRE infections (Narayanan et al., 2019). However, linezolid treatment is associated with several concerns. For example, linezolid treatment of VRE bloodstream infections has been linked with a mortality rate that can reach as high as 30%. Additionally, linezolid exhibits limited activity in decolonizing VRE from the GIT (Britt et al., 2017). Furthermore, linezolid treatment is associated with serious side effects, including bone marrow toxicity and neurotoxicity (Abou Hassan et al., 2016; Watkins, Lemonovich & File Jr, 2012). The combination of quinupristin/dalfopristin was previously approved by the FDA for treatment of VRE infections. However, this drug combination is rarely used due to concerns about toxicity (Dhanda et al., 2018). Daptomycin is another antibiotic that is frequently used in clinical practice as an anti-VRE treatment option (Baddour et al., 2015; Mermel et al., 2009). However, daptomycin is not approved by the FDA to treat VRE infections, and the lack of standard dosing for VRE infections is a concern (McKinnell & Arias, 2015). The serious threat of VRE is further compounded by the emergence of strains exhibiting resistance to linezolid, daptomycin, quinupristin/dalfopristin, and tigecycline (Donabedian et al., 2006; Fiedler et al., 2016; Gonzales et al., 2001; Munoz-Price, Lolans & Quinn, 2005). Furthermore, life-threatening infections caused by VRE, such as endocarditis and bloodstream infections, often require a β-lactam/aminoglycoside combination. However, most VRE strains are resistant to aminoglycosides and β-lactams, which compromises the treatment of these life-threatening infections (Arias, Contreras & Murray, 2010). Moreover, in addition to their intrinsic resistance to several antibiotics, VRE are able to develop resistance rapidly to multiple antibiotics via modification of the drug target or through horizontal gene transfer of transposons or plasmids carrying resistance elements (Mundy, Sahm & Gilmore, 2000). Consequently, the aforementioned reasons highlight the critical need to develop new, effective treatment options for VRE infections.

Drug repurposing is an efficient approach to drug discovery that saves both time and costs associated with drug innovation (AbdelKhalek et al., 2018; AbdelKhalek et al., 2019; Abutaleb & Seleem, 2020a; Abutaleb & Seleem, 2020b; Abutaleb & Seleem, 2020c; Brown, 2015; Mohammad et al., 2018; Younis et al., 2017). In an effort to meet the critical need for development of new, effective anti-VRE agents, we identified the FDA-approved carbonic anhydrase inhibitors (CAIs) acetazolamide, methazolamide and ethoxzolamide as promising anti-VRE agents (Younis et al., 2017). Additionally, through structure–activity relationship modifications to acetazolamide, our team developed acetazolamide analogs that exhibited potent in vitro activity against clinical isolates of VRE (Kaur et al., 2020). CAIs are FDA-approved drugs that suppress the activity of Carbonic anhydrase enzymes (CAs) and are clinically used as mild diuretics, anti-glaucoma medications, antiepileptics, and in the management of mountain sickness (Supuran, 2016). CAs act as catalysts hydrating carbon dioxide to bicarbonate and protons; this reaction constitutes the basis of regulation of pH in most living organisms (Supuran, 2020). Bacterial carbonic anhydrases (CAs) have recently garnered attention as bacterial targets for the development of novel antibacterial agents (Capasso & Supuran, 2015a; Supuran, 2011).

The aim of the current study was to evaluate the antimicrobial activity of dorzolamide both in vitro and *in vivo* against VRE. Dorzolamide is a CAI used to treat glaucoma (Ponticello et al., 1998). The antibacterial activity of dorzolamide was evaluated against a wide panel of clinical VRE strains. The in vitro killing kinetics of dorzolamide against VRE and the potential of dorzolamide to be combined with gentamicin were also investigated. Finally, the efficacy of dorzolamide in an in vivo VRE colonization reduction mouse model was evaluated.

## Materials and Methods

### Bacterial strains, media and chemicals

Enterococcal strains used in the study were obtained from the Biodefense and Emerging Infections Research Resources Repository (BEI Resources) (Manassas, VA, USA), and the American Type Culture Collection (ATCC) (Manassas, VA, USA). Media and reagents were purchased from commercial vendors: tryptic soy broth (TSB), tryptic soy agar (TSA), enterococcosel agar (Becton, Dickinson and Company, Cockeysville, MD, USA), and phosphate-buffered saline (PBS) (Corning, Manassas, VA, USA). Drugs used in the study were purchased commercially: dorzolamide (TCI America, Portland, OR, USA), linezolid and vancomycin (Chem-Impex International, Wood Dale, IL, USA), and ampicillin (IBI Scientific, Peosta, IA, USA).

### Antibacterial activity of dorzolamide against enterococci

MICs determination was performed utilizing the broth microdilution assay, as described before (CLSI, 2012). The MICs experiments were repeated at least 3 times. MICs reported are the lowest drug concentrations that completely inhibited the bacterial growth, as observed visually. MIC_50_ and MIC_90_ are the lowest concentration of each drug that inhibited the growth of 50% and 90% of the tested isolates, respectively.

### Killing kinetics of dorzolamide against VRE

A time-kill assay was performed for dorzolamide and linezolid against *E. faecium* HM-952, following a method described previously (Abutaleb & Seleem, 2020a; Abutaleb & Seleem, 2020c).

### Combination testing of dorzolamide with gentamicin against VRE

To evaluate the interactions between dorzolamide and gentamicin against VRE clinical isolates, a standard checkerboard assay was utilized (Abutaleb & Seleem, 2020c; MartinezIrujo et al., 1996; Mohammad, Cushman & Seleem, 2015). The fractional inhibitory concentration indices (FICIs) were calculated using the following equation: FICI = FICI_A_ + FICI_B_ = (C_A_/MIC_A_) + (C_B_/MIC_B_), where MIC_A_ and MIC_B_ are the MICs of drugs A and B alone, respectively, and C_A_ and C_B_ are the MICs of the two drugs in combination, respectively. Interactions where the FICI was ≤0.5 were categorized as synergistic (SYN). An FICI value of >0.5 − 1.25 was considered additive (ADD), an FICI value of >1.25 − 4 was considered indifferent, and FICI values of >4 were considered antagonistic (Eldesouky et al., 2020; Meletiadis et al., 2010).

### In vivo VRE colonization reduction mouse model

All animal housing and experiments were reviewed, approved and performed under the guidelines of the Purdue University Animal Care and Use Committee (protocol number 1905001908) and carried out in strict accordance with the recommendations in the Guide for the Care and Use of Laboratory Animals of the National Institutes of Health. Mice were housed in individually ventilated cages (5 per cage, 12 h light/dark cycle, in the animal facility) with free access to food and water. All mice were acclimatized for seven days before any experimental procedure. The VRE colonization reduction murine model, described previously (AbdelKhalek et al., 2018; Mohammad et al., 2018), was performed to evaluate the ability of dorzolamide to reduce the VRE burden present in the GIT of mice. Briefly, 8-week-old female C57BL/6 mice (obtained from Jackson laboratories, ME, USA) were sensitized with 0.5 g/l ampicillin in drinking water, for 7 days before being infected with 1.3 × 10^8^ CFU/mL of *E. faecium* HM-952 via oral gavage. Seven-days post-infection, mice were randomly allocated into three groups (*n* = 5/each) for treatment via oral gavage: one group for dorzolamide (10 mg/kg), one group for linezolid (10 mg/kg), and one group for the vehicle (10% DMSO:90% PBS) (negative control). Treatments were continued quaque die (q.d.) for eight consecutive days. The VRE colonization reduction model does not involve expected mice mortality throughout the experiment. However, certain criteria for exclusion and euthanizing the animals prior to the planned end of the experiment were established. Any animal that meets any two of the group I criteria (a. rough coat and unkempt, b. eyes are full or partially closed for 10 minutes, c. markedly diminished resistance to being handled (grimace response), d. markedly decreased movement/lethargy, e. hunched posture, and f. distended abdomen), will be excluded and euthanized. Any mouse having one of group II criteria (a. inability to eat or drink, b. moribund/unresponsive, c. failure to right itself when placed on its back, d. dyspnea, or e. 15% or more loss in the body weight) will be euthanized. Treatments were administered daily on the same arrangement and at the same time, and cages locations were kept at the same positions throughout the experiment to minimize confounders.

Mice fecal pellets were aseptically collected on days 0 (before treatment) and days 3, 5 and 7 (after the start of treatment). Thereafter, all mice were euthanized humanely, via carbon dioxide asphyxiation, and their cecal and ileal tissues were aseptically collected (all mice were included in the analysis). Fecal pellets and cecal and ileal contents were diluted in PBS and plated on enterococcosel agar plates containing 8 µg/mL vancomycin. Plates were incubated at 37 °C for 48 hours before determining the bacterial CFU present in each sample. The data of CFU counts in fecal contents were analyzed via two-way ANOVA with post hoc Dunnett’s test for multiple comparisons (*P* < 0.05), while that of CFU counts in cecal and ileal contents were analyzed via one-way ANOVA with post hoc Dunnett’s test for multiple comparisons (*P* < 0.05). Asterisks (*) denote statistically significant difference between the results obtained for dorzolamide or linezolid in comparison to the negative control group (vehicle). Pounds (#) denote statistically significant difference between the results obtained for dorzolamide in comparison to linezolid.

### Statistical analyses

GraphPad Prism version 8.0 for Windows (GraphPad Software, La Jolla, CA, USA) was used to conduct the statistical analyses presented in this study. The time kill assay results and data obtained from fecal samples were analyzed via two-way ANOVA with post hoc Dunnett’s test for multiple comparisons. The data obtained from cecal and ileal contents were analyzed via one-way ANOVA with post hoc Dunnett’s test for multiple comparisons.

## Results

### Dorzolamide exhibits potent in vitro activity against strains of VRE

The antibacterial activity of dorzolamide was evaluated against a panel of 29 enterococcal strains that included 23 clinical VRE strains. As presented in Table 1, dorzolamide exhibited potent in vitro activity against all enterococcal strains tested. Dorzolamide inhibited growth of enterococcal isolates at concentrations that ranged from 1 µg/mL to 8 µg/mL. Dorzolamide, at 4 µg/mL, inhibited growth of both 50% (MIC_50_) and 90% (MIC_90_) of enterococcal isolates. Moreover, dorzolamide’s MIC values were similar when tested against strains of VRE, vancomycin-sensitive *E. faecalis* and *E. faecium* strains, and the linezolid-resistant *E. faecium* NR-31903 strain. Linezolid, at 1 µg/mL, inhibited 50% and 90% of the enterococcal strains tested.

**Table 1:** MICs (µg/mL) of dorzolamide against clinical enterococcal isolates.

**Enterococcal strain**	**Dorzolamide**	**Linezolid**	**Vancomycin**
*E. faecium* NR-28978	4	1	>128
*E. faecium* NR-31903	4	16	>128
*E. faecium* NR-31909	4	1	>128
*E. faecium* NR-31912	4	0.5	>128
*E. faecium* NR-31914	4	1	>128
*E. faecium* NR-31915	4	1	>128
*E. faecium* NR-31916	4	0.5	128
*E. faecalis* NR-31971	4	1	64
*E. faecalis* NR-31972	2	1	>128
*E. faecium* NR-32052	4	0.5	>128
*E. faecium* NR-32053	4	0.5	>128
*E. faecium* NR-32054	4	0.5	128
*E. faecium* NR-32065	1	0.25	>128
*E. faecium* NR-32094	8	0.5	>128
*E. faecalis* HM-201	4	1	>128
*E. faecalis* HM-334	2	1	>128
*E. faecalis* HM-335	2	0.5	>128
*E. faecalis* HM-934	4	1	>128
*E. faecium* HM-952	4	1	>128
*E. faecium* HM-965	2	0.5	>128
*E. faecium* HM-968	4	1	>128
*E. faecium* HM-970	4	1	>128
*E. faecium* ATCC 700221	1	0.5	>128
*E. faecium* NR-31933	8	1	4
*E. faecium* NR-31935	4	1	1
*E. faecium* NR-31937	8	1	2
*E. faecium* NR-31954	4	1	2
*E. faecalis* NR-31970	4	1	1
*E. faecalis* NR-31975	4	1	1
**MIC**_**50**_	**4**	**1**	**>128**
**MIC**_**90**_	**4**	**1**	**>128**

**Notes.**

**MIC_50_:** The concentration of the test agent that inhibited growth of 50% of the tested strains

**MIC_50_:** The concentration of the test agent that inhibited growth of 90% of the tested strains

### Dorzolamide exhibits a bacteriostatic effect against VRE

**Figure 1:**
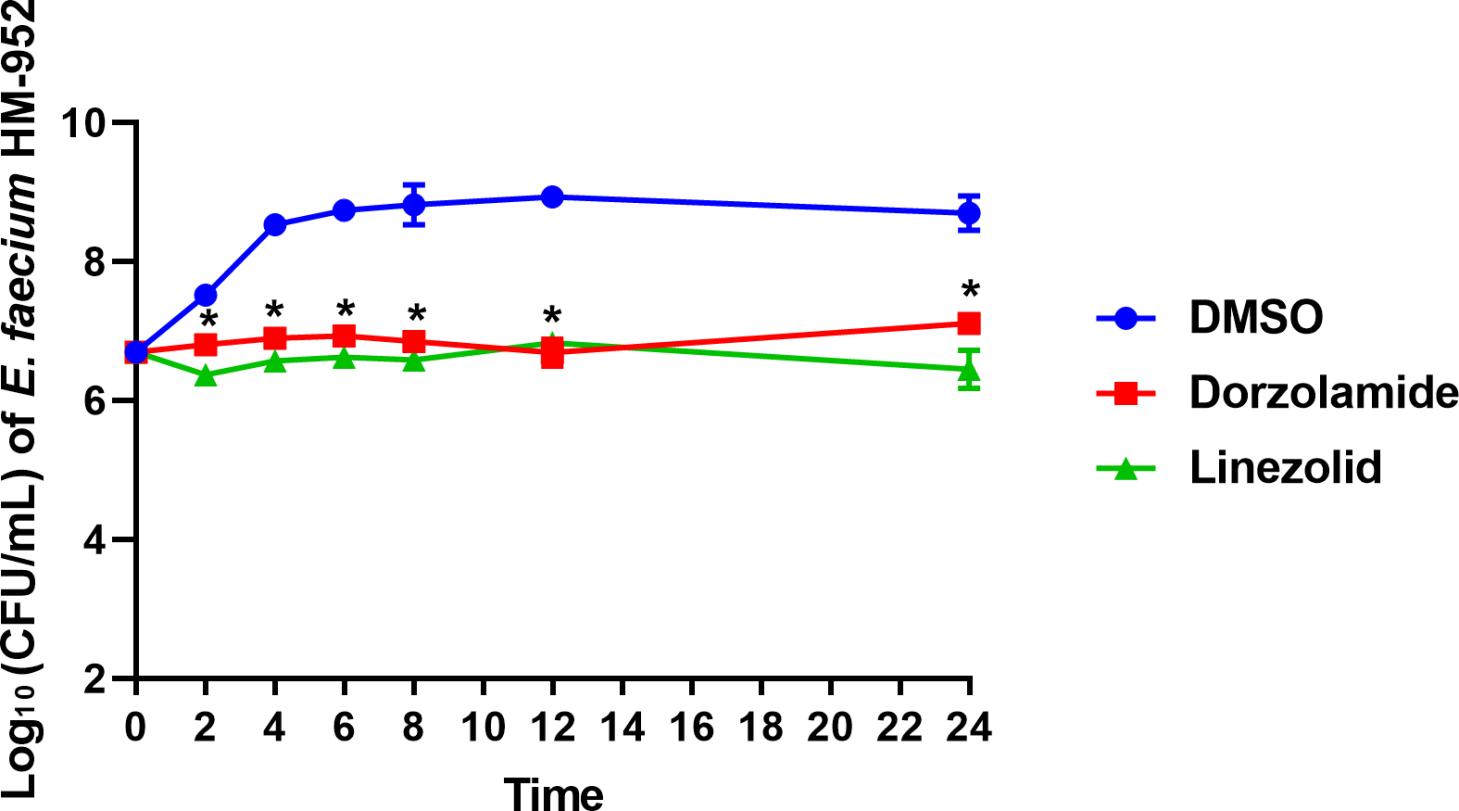
Time-kill assay of dorzolamide and linezolid (tested in triplicates, at 10 × MIC) against *E. faecium* HM-952. DMSO (vehicle) served as a negative control. The error bars represent standard deviation values for each drug studied. The data were analyzed via a two-way ANOVA with post-hoc Dunnett’s test for multiple comparisons. An asterisk (*) indicates a statistically significant difference (*P* < 0.05) between dorzolamide or linezolid treatment compared to DMSO treatment (negative control).

To determine if dorzolamide exhibits a bactericidal or bacteriostasis effect in vitro against VRE, a time-kill assay was conducted. As presented in Fig. 1, in the presence of dorzolamide (at 10 × MIC), the bacterial count of *E. faecium* HM-952 remained almost constant over 24 hours but was significantly reduced as compared to the negative control (DMSO).

### Dorzolamide exhibits a synergistic interaction with gentamicin against VRE isolates

Using the standard checkerboard assay, we investigated whether the combination of an aminoglycoside (gentamicin) with dorzolamide could enhance the activity of gentamicin against VRE. As presented in Table 2, dorzolamide exhibited a synergistic interaction with gentamicin against 4 out of 10 tested strains, with a fractional inhibitory concentration index (FICI) that ranged from 0.31 to 0.50. The dorzolamide/gentamicin combination demonstrated an additive relationship against 6 of the tested strains. Remarkably, in the presence of 0.5 × MIC of dorzolamide, the MIC values of gentamicin were reduced significantly in 4 of these strains. The MIC of gentamicin improved from 32 µg/mL to 4 µg/mL in two strains, from 64 µg/mL to 4 µg/mL in one strain, and from 128 µg/mL to 2 µg/mL in one strain.

**Table 2:** MICs (µg/mL) of dorzolamide and gentamicin tested alone and in combination against VRE clinical isolates.

**VREstrain**	**MIC (µg/mL)**				**FICI**^a^	Interaction^*^
	**Dorzolamide**		**Gentamicin**			
	**Alone**	**Combined with gentamicin**	**Alone**	**Combined with dorzolamide**		
*E. faecium* NR-31912	4	1	64	16	0.50	SYN
*E. faecium* NR-31915	4	1	16	4	0.50	SYN
*E. faecium* NR-31916	4	2	32	4	0.63	ADD
*E. faecalis* NR-31971	4	1	256	32	0.38	SYN
*E. faecalis* NR-31972	2	1	512	64	0.63	ADD
*E. faecalis* HM-934	4	1	64	4	0.31	SYN
*E. faecalis* HM-201	4	2	512	32	0.56	ADD
*E. faecalis* HM-335	2	1	512	16	0.53	ADD
*E. faecium* HM-968	4	2	128	2	0.52	ADD
*E. faecium* HM-970	4	2	32	4	0.63	ADD

**Notes.**

a FICI, fractional inhibitory concentration index

* FICI < 0.5 was considered synergistic (SYN); 0.5 < FICI ≤ 1.25 was considered additive (ADD); 1.25 < FICI ≤ 4 was considered indifferent; FICI > 4 was considered antagonistic.

### Dorzolamide significantly reduced the burden of VRE in the GIT in a colonization reduction murine model

**Figure 2:**
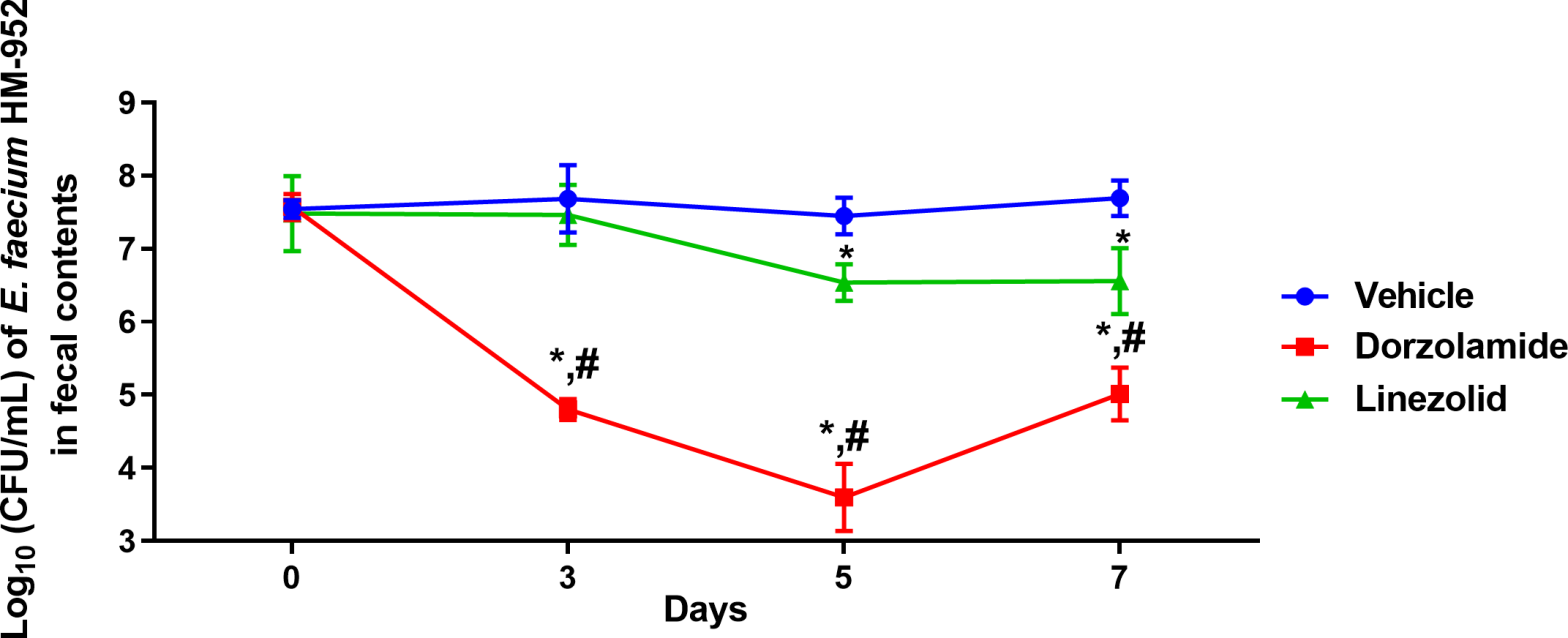
Log_10_ (CFU/mL) of vancomycin-resistant *E*. *faecium* HM-952 in the fecal contents of infected mice. Mice were orally treated once daily for 8 days with each drug. Fecal samples were collected from each group of mice on day 0 (before the start of treatment) and on days 3, 5 and 7 (post-treatment) and VRE colonies were counted. The CFU data were analyzed via a two-way ANOVA with post-hoc Dunnett’s test for multiple comparisons. An asterisk (*) indicates a statistically significant difference (*P* < 0.05) between mice treated with dorzolamide or linezolid compared to the vehicle (negative control). A pound sign (#) indicates a statistically significant difference (*P* < 0.05) between mice treated with dorzolamide compared to linezolid.

**Figure 3:**
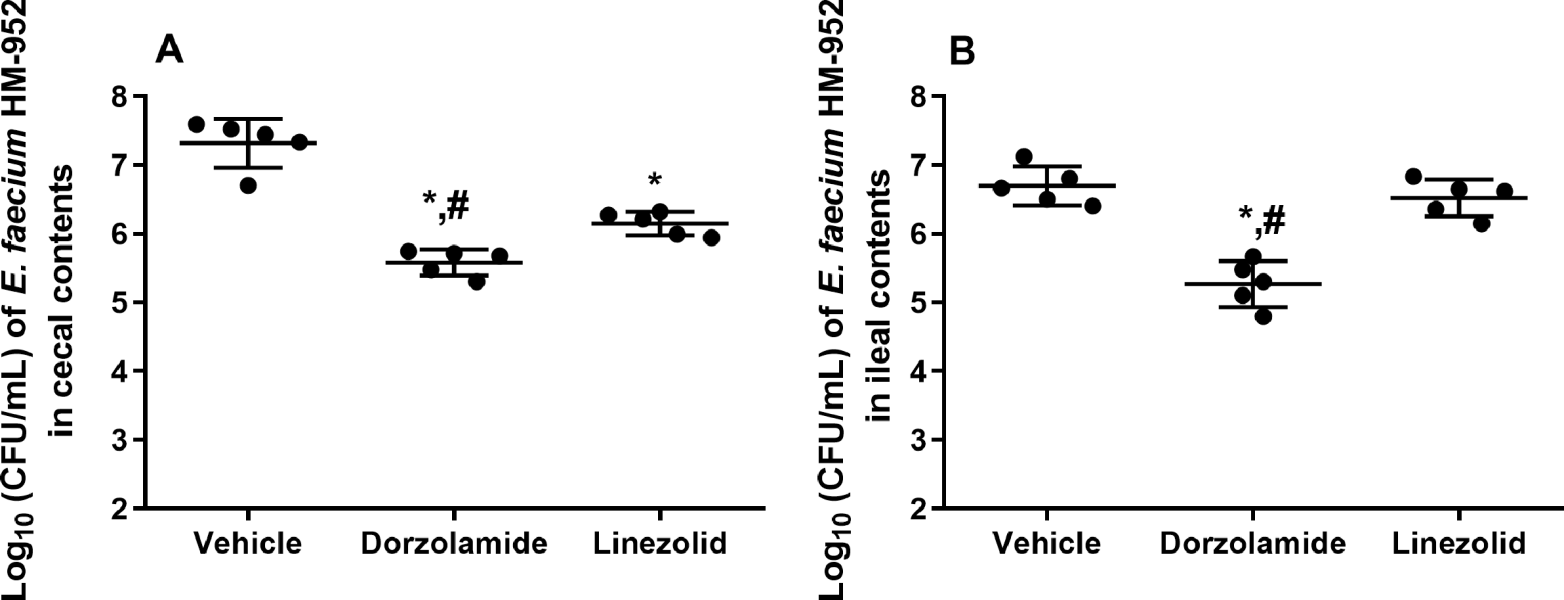
Log_10_ (CFU/mL) of vancomycin-resistant *E*. *faecium* HM-952 in: (A) the cecal contents of infected mice, and (B) the ileal contents of infected mice. Mice were orally treated once daily for 8 days with each drug. Mice ceca and ilea were aseptically removed from each group after euthanasia, diluted, and counted. The data were analyzed via a one-way ANOVA with post-hoc Dunnett’s test for multiple comparisons. An asterisk (*) indicates a statistically significant difference (*P* < 0.05) between mice treated with dorzolamide or linezolid compared with vehicle-treated mice. A pound sign (#) indicates a statistically significant difference (*P* < 0.05) between mice treated with dorzolamide compared to linezolid-treated mice.

Next, we evaluated dorzolamide’s ability to decrease the burden of VRE in mice intestinal tissues in a VRE decolonization mouse model. Dorzolamide was found to be superior to linezolid in the mouse study (Figs. 2 and 3). After only 3 days of treatment, dorzolamide (10 mg/kg) significantly reduced the burden of VRE in mice fecal samples by 2.9-log_10_ (99.9% reduction). In contrast, linezolid (10 mg/kg) did not reduce the burden of VRE in mice fecal samples (Fig. 2). The burden of VRE continued to decrease with dorzolamide treatment, resulting in a 3.86-log_10_(99.99%) reduction after 5 days. Dorzolamide significantly outperformed linezolid (0.91-log_10_ reduction) in reducing the burden of VRE in fecal samples after 5 days of treatment. After 7 days of dorzolamide treatment, the count of VRE slightly increased (compared to day 5) resulting in a 2.69-log_10_ (99.8%) reduction compared to vehicle-treated mice. Dorzolamide’s reduction of VRE in fecal samples significantly surpassed the 1.1-log_10_ (92%) reduction in VRE CFU observed with linezolid after 7 days of treatment (Fig. 2). Notably, the VRE count in the fecal samples of the vehicle-treated group remained in the range of 10^7^ CFU/mL during the experiment. This indicates that the decrease in VRE burden observed in the dorzolamide- or linezolid-treated mice was mainly due to the treatments received.

Furthermore, VRE burden in the cecal and ileal tissues of mice, after euthanasia, was determined. Dorzolamide significantly reduced the VRE count in the cecal and ileal contents of mice. In the cecal contents, dorzolamide decreased VRE burden by 1.74-log_10_ (98.2% reduction). Linezolid decreased the VRE burden in the cecal contents by 1.2-log_10_ (93.2% reduction) (Fig. 3). In the ileal contents of mice, dorzolamide significantly reduced VRE burden compared to linezolid. Dorzolamide treatment resulted in a 1.5-log_10_(96.3%) reduction in VRE compared to vehicle-treated mice. In contrast, linezolid did not reduce VRE burden in the ileal contents of mice (Fig. 3).

## Discussion

Vancomycin-resistant enterococci are a leading cause of nosocomial infections, but the therapeutic options available for treatment of these infections are limited (Lebreton et al., 2013). VRE are responsible for more than one third of infections caused by all enterococci and over 5% of all deaths attributed to an antibiotic-resistant bacterial infection in the U. S. are due to VRE (Mohammad et al., 2018). VRE are capable of overgrowing the body’s normal flora in the gastrointestinal tract, particularly after the administration of broad-spectrum antibiotics. After colonizing the GIT, VRE can translocate across human epithelial cells, which leads to systemic infections such as septicemia, UTI, endocarditis, and surgical site infections (Ubeda et al., 2010). Given the dearth of effective therapeutic options and increasing resistance to the available treatment options, there is an urgent need to develop new therapeutics to treat VRE infections.

We recently identified carbonic anhydrase inhibitors, namely acetazolamide and its analogs, as potent inhibitors of VRE (Kaur et al., 2020; Younis et al., 2017). Carbonic anhydrases from different bacteria such as *Neisseria gonorrhoeae*, *Helicobacter pylori*, *Mycobacterium tuberculosis*, *Streptococcus pneumoniae*, *Brucella spp*, and *Vibrio cholerae* have been successfully cloned and characterized (Supuran, 2011). In addition, genes encoding for carbonic anhydrases have been annotated in the *E. faecalis* genome (Capasso & Supuran, 2015b; Smith et al., 1999). Consequently, bacterial carbonic anhydrases have recently garnered attention as promising microbial targets for development of new antimicrobials (Capasso & Supuran, 2015a; Supuran, 2011; Supuran & Capasso, 2020). For instance, a β-carbonic anhydrase present in *Helicobacter pylori* was shown to be a possible target for gastric drugs and dorzolamide was reported as one of the inhibitors for *H. pylori* carbonic anhydrase (Nishimori et al., 2007; Nishimori et al., 2006). In this vein, the carbonic anhydrase inhibitors, ethoxzolamide, acetazolamide, and methazolamide were reported to inhibit *H. pylori in* vitro, with ethoxzolamide exhibiting the most potent activity (Modak et al., 2019). Additionally, sulfonamide carbonic anhydrase inhibitors such as acetazolamide, methazolamide, diclofenamide, dorzolamide, brinzolamide, and benzolamide were shown to exhibit submicromolar inhibition against *M*. tuberculosis. Ethoxzolamide also showed efficacy in *M.* tuberculosis-infected macrophages and mice suggesting that mycobacterial β-CAs perform very important roles in mycobacterial infections and present themselves as important drug target (Johnson et al., 2015). Moreover, our group recently reported a drug-repurposing and optimization study for acetazolamide-based VRE inhibitors, and our data suggested the intracellular targets for the molecules are likely putative *α*-carbonic and *γ*-carbonic anhydrases, and homology modeling and molecular dynamics simulations were performed (Kaur et al., 2020).

This study aimed to investigate the activity of dorzolamide (an FDA-approved CAI) against VRE both in vitro and *in vivo.* Dorzolamide was tested against 23 clinical VRE strains. It exhibited potent in vitro inhibitory activity against all 23 strains tested (MIC values ranged from 1 µg/mL to 8 µg/mL). Moreover, dorzolamide effectively inhibited growth of both vancomycin-resistant *E*. *faecium* and *E*. *faecalis* strains, unlike the combination of quinupristin/dalfopristin, which is reported to be less efficacious against *E. faecalis* strains (Hollenbeck & Rice, 2012). In addition, the MIC values of dorzolamide were consistent against both vancomycin-resistant and vancomycin-sensitive strains. Moreover, we determined that dorzolamide exhibits a bacteriostatic effect against VRE in vitro, which is similar to linezolid (AbdelKhalek et al., 2018; Mohammad et al., 2018).

One of the major challenges in treating enterococcal infections with a single agent is that it often provides a bacteriostatic effect, even with drugs which are typically bactericidal, such as β-lactams (Baddour et al., 2015; Brown Gandt et al., 2018). Accordingly, current guidelines recommend combination therapy of a β-lactam and an aminoglycoside (to exert bactericidal activity) to treat systemic infections caused by enterococci, particularly endocarditis (Baddour et al., 2015). However, many enterococcal strains are relatively impermeable to aminoglycosides, and enterococcal resistance to aminoglycosides is prevalent (Chow, 2000). As a consequence, the concentration of aminoglycosides necessary to kill VRE could be higher than their clinically achievable concentration (Brown Gandt et al., 2018; Murray, 1990). Consequently, we evaluated the combination of dorzolamide with the aminoglycoside gentamicin against 10 VRE strains. A checkerboard assay found synergistic interactions between dorzolamide and gentamicin against 4 strains of VRE and an additive effect against six strains of VRE. Interestingly, dorzolamide resensitized some tested VRE strains to gentamicin reducing its MIC by 8- to 64-fold. Therefore, using dorzolamide in combination with gentamicin could potentially decrease the dose of gentamicin administered to patients clinically. Using a lower treatment dose is highly desirable in the treatment of systemic VRE infections, especially in patients with comorbid conditions.

Finally, our study investigated dorzolamide’s effect in an in vivo VRE colonization reduction murine model. Enterococci normally inhabit the human GIT and remain under the control of the normal flora present in the gut. Disturbance of the normal flora balance can lead to VRE overgrowth and colonization of the gut. VRE can subsequently spread throughout the body causing serious infections including endocarditis, bloodstream infections, and UTIs (Ubeda et al., 2010). In addition, dysbiosis and colonization by VRE was found to exacerbate irritable bowel disorders such as Crohn’s disease (Seishima et al., 2019; Steck et al., 2011; Zuo & Ng, 2018). Thus, suppressing VRE colonization of the GIT is considered an alternative strategy to curb VRE infections, particularly in highly-susceptible people such as immunocompromised patients, organ transplant recipients, and patients in intensive care units (Mohammad et al., 2018; Wong et al., 2001). Though enterococcal colonization of the GIT contributes to the development of systemic infections, there is no effective drug currently approved for enterococcal decolonization (Ubeda et al., 2010). Linezolid, the only FDA-approved antibiotic to treat VRE infections, is ineffective as a VRE decolonizing agent, which could be attributed to its rapid absorption from the GIT. Consequently, the need to develop new agents that can successfully decolonize VRE from the GIT cannot be overemphasized. Although both dorzolamide and linezolid exhibited bacteriostatic activity against VRE in vitro, dorzolamide was superior to linezolid in reducing the burden of VRE in the GIT of infected mice in our mouse model. This result suggests that agents exhibiting bacteriostatic activity in vitro could be effective decolonizing agents and should not be excluded from consideration. Linezolid, in accordance with previous reports (AbdelKhalek et al., 2018; Mohammad et al., 2018), exhibited lower activity in reducing the burden of VRE in the GIT of infected mice. The limited effect of linezolid in reducing the burden of VRE in the GIT could be due to several reasons such as linezolid’s (1) rapid absorption from the GIT (Beringer et al., 2005), (2) low concentration in the stool (Lode et al., 2001), or (3) limited activity against a high bacterial inoculum (∼10^8^ CFU), as is the case for VRE colonization of the GIT (Pultz, Stiefel & Donskey, 2005). Although dorzolamide proved to be effective in the VRE colonization reduction mouse model, a future investigation will need to investigate whether dorzolamide has any deleterious impact on the gut microbiota.

It is worth mentioning that dorzolamide is a very safe drug. It was reported that its oral LD_50_ is very high (1,927 mg/kg), and (1,320 mg/kg) in rats and mice, respectively while its subcutaneous LD_50_ is >2g/kg in mice and rats (BritishPharmacopeia). It does not produce acid–base imbalance or electrolyte disturbances. It is also, not associated with severe systemic adverse effects and can be administered to patients with severe respiratory diseases and heart diseases (Balfour & Wilde, 1997; Kobayashi & Naito, 2000).

## Conclusions

In conclusion, the current study presents dorzolamide as a new drug for treatment of VRE infections. Dorzolamide exhibited a potent in vitro inhibitory activity against enterococci. Additionally, dorzolamide interacted synergistically with gentamicin, reducing its MIC values to low clinically achievable concentrations. Moreover, dorzolamide outperformed linezolid in an in vivo VRE colonization reduction mouse model. The results altogether suggest that, dorzolamide represents a promising novel therapeutic option for the treatment of VRE infections.

##  Supplemental Information

10.7717/peerj.11059/supp-1 Supplemental Information 1 Completed full ARRIVE 2.0 checklist

10.7717/peerj.11059/supp-2 Supplemental Information 2 Raw data for time kill assay

10.7717/peerj.11059/supp-3 Supplemental Information 3 Raw data for fecal contents counts

10.7717/peerj.11059/supp-4 Supplemental Information 4 Raw data for cecal contents counts

10.7717/peerj.11059/supp-5 Supplemental Information 5 Raw data for ileal contents counts

## Additional Information and Declarations

### Data Availability

The following information was supplied regarding data availability: Raw measurements are available in the Supplemental Files.
